# Clinical Outcomes of Ketamine Therapy for Depression, Anxiety, and PTSD in Post-TBI Patients

**DOI:** 10.1192/j.eurpsy.2026.11400

**Published:** 2026-07-31

**Authors:** W. Aldulaimy, A. Shah

**Affiliations:** 1 Psychiatry; 2 Texas Institute for Graduate Medical Education and Research, San Antonio, United States

## Abstract

**Introduction:**

TBI patients often suffer from severe, treatment-resistant depression and PTSD. Ketamine shows promise in non-TBI populations, but its efficacy in TBI patients needs exploration for efficacy and tolerability.

**Objectives:**

Evaluate ketamine’s clinical effectiveness and safety (PHQ-9, GAD-7, PCL-5 scores) in post-TBI patients, comparing IM, intranasal, and oral routes.

**Methods:**

Design: Retrospective chart review of 35 adult TBI patients (mean age 38.4; TBI 3.5 years prior).

Formulations: IM (0.5-0.75 mg/kg), Intranasal (84 mg esketamine), and Oral Dissolving Tablets (100-300 mg).

Measures: PHQ-9, GAD-7, and PCL-5 scores.

Analysis: Efficacy by % reduction, response (≥50% reduction), and remission (PHQ-9 < 5, PCL-5 < 20).

**Results:**

Magnitude: Large effect sizes observed (32.7% GAD-7/ODT to 65.6% PCL-5/Nasal). Clinical: 72% response, 45% remission rates. Route-Dependent: Parenteral (IM/Nasal) outperformed ODT due to better bioavailability. Rapid Onset: Initial improvement within 5-10 days. Safety: Favorable profile with mild, transient adverse events.

**Conclusions:**

Magnitude: Large effect sizes observed (32.7% GAD-7/ODT to 65.6% PCL-5/Nasal). Clinical: 72% response, 45% remission rates. Route-Dependent: Parenteral (IM/Nasal) outperformed ODT due to better bioavailability. Rapid Onset: Initial improvement within 5-10 days. Safety: Favorable profile with mild, transient adverse events. 1. Directions

**Disclosure of Interest:**

None Declared

